# Berberine Reduces Lipid Accumulation by Promoting Fatty Acid Oxidation in Renal Tubular Epithelial Cells of the Diabetic Kidney

**DOI:** 10.3389/fphar.2021.729384

**Published:** 2022-01-05

**Authors:** Qingfeng Rong, Baosheng Han, Yafeng Li, Haizhen Yin, Jing Li, Yanjuan Hou

**Affiliations:** ^1^ Department of Endocrinology, Second Hospital, Shanxi Medical University, Taiyuan, China; ^2^ Department of Cardiac Surgery, Shanxi Cardiovascular Hospital, Taiyuan, China; ^3^ Department of Nephrology, Shanxi Province People’s Hospital, Taiyuan, China; ^4^ Shanxi Provincial Key Laboratory of Kidney Disease, Taiyuan, China; ^5^ Central Laboratory, Shanxi Province People’s Hospital, Taiyuan, China; ^6^ Department of Nephrology, Second Hospital, Shanxi Medical University, Taiyuan, China

**Keywords:** diabetic kidney disease, renal tubular epithelial cells, fatty acid oxidation, mitochondria, berberine

## Abstract

Abnormal lipid metabolism in renal tubular epithelial cells contributes to renal lipid accumulation and disturbed mitochondrial bioenergetics which are important in diabetic kidney disease. Berberine, the major active constituent of *Rhizoma coptidis* and *Cortex phellodendri*, is involved in regulating glucose and lipid metabolism. The present study aimed to investigate the protective effects of berberine on lipid accumulation in tubular epithelial cells of diabetic kidney disease. We treated type 2 diabetic db/db mice with berberine (300 mg/kg) for 12 weeks. Berberine treatment improved the physical and biochemical parameters of the db/db mice compared with db/m mice. In addition, berberine decreased intracellular lipid accumulation and increased the expression of fatty acid oxidation enzymes CPT1, ACOX1 and PPAR-α in tubular epithelial cells of db/db mice. The mitochondrial morphology, mitochondrial membrane potential, cytochrome c oxidase activity, mitochondrial reactive oxygen species, and mitochondrial ATP production in db/db mice kidneys were significantly improved by berberine. Berberine intervention activated the AMPK pathway and increased the level of PGC-1α. *In vitro* berberine suppressed high glucose-induced lipid accumulation and reversed high glucose-induced reduction of fatty acid oxidation enzymes in HK-2 cells. Importantly, in HK-2 cells, berberine treatment blocked the change in metabolism from fatty acid oxidation to glycolysis under high glucose condition. Moreover, berberine restored high glucose-induced dysfunctional mitochondria. These data suggested that berberine alleviates diabetic renal tubulointerstitial injury through improving high glucose-induced reduction of fatty acid oxidation, alleviates lipid deposition, and protect mitochondria in tubular epithelial cells.

## Introduction

Diabetic kidney disease (DKD), a common and severe chronic complication of diabetes mellitus, especially type 2 diabetes mellitus, has become a worldwide public health problem (Fineberg et al., 2013). Epidemiologically, chronic renal failure caused by DKD accounted for 27.7% of the incidence of chronic renal failure (Koye et al., 2018). Significant financial resources are spent annually to treat these patients. However, the pathogenesis of DKD is unclear, and the treatment of DKD requires improvement.

Initially, DN is considered to be a progressive disease characterized by albuminuria and an increase in GFR, both considered markers of glomerular damage. However, Recent clinical studies have demonstrated that the ‘non-albuminuric phenotype’ is now becoming the predominant mode of DN presentation (Piscitelli et al., 2017; Viazzi et al., 2017). As a result, emerging evidence supporting a role for tubular involvement in DKD, renal tubular epithelial cells (TECs) may play a role as an initiator, driver or contributor in the early pathogenesis of diabetes affecting the kidney (Russo et al., 2009; Tang and Lai, 2012; Zeni et al., 2017; Gonçalves-Dias et al., 2019). Tubular cells are also the primary targets of elevated blood glucose and lipid levels, which contribute to DKD initiation and progression. Tubule epithelial lipid accumulation has received substantial attention, especially in type 2 DKD, which is characterized by metabolic disorder (Reidy et al., 2014). Excess accumulation of triglycerides induces cellular lipotoxicity, contributing to the development of tubulointerstitial fibrosis (Stadler et al., 2015). Lipid homeostasis in cells is regulated by a tight balance among fatty acid uptake, oxidation, and synthesis. Normally, TECs have one of the highest energy demands in the kidney, relying on fatty acid oxidation (FAO) as their energy source (Weinberg, 2006). However, in DKD, TECs display increased glycolysis and reduced mitochondrial FAO (Forbes and Thorburn, 2018). The levels of enzymes and regulators of FAO were reduced in TECs from patients with DKD and in animal models, which contribute to intracellular lipid accumulation, dysfunctional mitochondria, and the development of tubulointerstitial fibrosis (Weinberg, 2006; Kang et al., 2015; Stadler et al., 2015; Forbes and Thorburn, 2018). Restoring normal FAO levels via drugs or other strategies is beneficial to treat DKD (Forbes and Thorburn, 2018). Therefore, exploration of natural compounds that could enhance FAO and protect mitochondrial function has attracted increasing interest.

Berberine (BBR; [C_20_H_18_NO_4_]^+^), an isoquinoline alkaloid with a long history of medicinal application, is the major active constituent of *Rhizoma coptidis* and *Cortex phellodendri* (Zhang et al., 2008; Afshinnia et al., 2018). BBR has multiple pharmacological activities, for example, lowering blood glucose, regulating blood lipids, antioxidant activity, anti-inflammatory activity, and increasing insulin sensitivity, and thus might represent therapeutic drug to treat DKD (Zhang et al., 2008; Ni et al., 2015; Afshinnia et al., 2018). Furthermore, BBR promotes mitochondrial energy output and FAO by targeting peroxisome proliferator-activated receptor gamma coactivator 1-alpha (PGC-1α) and stimulates AMP-activated protein kinase (AMPK) signaling in podocytes in DKD (Li et al., 2014). Importantly, BBR protects human TECs from ischemia/reperfusion injury by inhibiting mitochondrial stress (Qin et al., 2020). However, the mechanisms underlying BBR’s reno-protective effects are unclear and whether BBR could promote FAO in TECs of the diabetic kidney is unknown. Therefore, the present study aimed to investigate protective effects and mechanisms of BBR against renal injury in type 2 diabetic mice.

## Materials and Methods

### Experimental Animals

Adult male (6–8 weeks old, *n* = 8) C57BLKS/J db/db diabetic and db/m normal male mice were purchased from the Model Animal Research Center of Nanjing University. All experimental animals were housed in a temperature-controlled room in the animal center of the Shanxi Medical University. They were given free access to food and water. BBR was provided by Sigma-Aldrich (St. Louis, MO, United States). After an 1-week acclimatization period, db/db diabetic and db/m normal mice were randomly subdivided into either a BBR subgroup (*n* = 8) (administered at 300 mg/kg/d) or vehicle (*n* = 8) (0.9% saline of the same volume). The BBR dose was based on related studies showing the efficacy of BBR without adverse effects (Li et al., 2014). Treatments were administered via gavage for 12 weeks. Body weight and blood glucose was monitored weekly. At the end of the experiments, the mice were fasted for 6 h and blood samples were obtained from the abdominal aorta to determine renal function, blood glucose, and blood lipids. Kidney tissues and 24-h urine samples were collected from each mouse for further study. All experimental protocols were conducted according to the guidelines of the Ethics Review Committee for Animal Experimentation of Shanxi Medical University.

### Cell Culture

The human renal proximal tubular cell line (HK-2 cells) was obtained from the ATCC (American Type Culture Collection, Manassas, VA, United States) and maintained in Dulbecco’s modified Eagle’s medium (DMEM) containing 1 g/L glucose supplemented with 10% fetal bovine serum (FBS), 100 U/mL penicillin, and 100 mg/ml streptomycin at 37°C in a 5% CO_2_ atmosphere. D-glucose, palmitic acid (PA) were purchased from Sigma. HK-2 cells were serum starved in 0.2% serum containing DMEM (1 g/L glucose) for at least 12 h before stimulation with normal glucose (NG, 5.6 mM), NG plus BBR (NG + BBR, 30 µM), high glucose (HG, 30 mM), HG plus BBR (HG + BBR, 30 µM) according to the experimental design.

### Metabolic Data

At the end of the experiments, the mice were moved to metabolic cages individually. Urine was collected over a 24 h period. Urinary albumin, fasting blood glucose (FBG), serum creatinine (Scr), and serum lipid profiles, including free fatty acid (FFA), triglyceride (TG), total cholesterol (TC), low density lipoprotein (LDL) cholesterol, and high density lipoprotein (HDL) cholesterol content, were measured using an Olympus AU2700 Analyzer (Olympus, Tokyo, Japan).

### Renal Pathology and Immunohistochemistry

Renal tissues were fixed using 4% paraformaldehyde, dehydrated, embedded in paraffin, and sectioned as 4 μm and 2 μm-thick sections. Sections (2 μm) were used for periodic acid-Schiff (PAS). The glomerular and tubulointerstitial injury index was conducted by a pathologist in a blinded fashion, as described previously (Tervaert et al., 2010; Hou et al., 2018). The glomerular injury index was graded from 0 to 4 on the basis of the degree of glomerulosclerosis and mesangial matrix expansion: grade 0 represented normal glomeruli; grade 1 represented a mesangial matrix expansion area up to 25%; grade 2 represented mesangial matrix expansion of >25–50%; grade 3 represented mesangial matrix expansion of >50–75%; and grade 4 represented>75% mesangial matrix expansion. The tubular area consisting of ∼80 ± 100 proximal tubules in each mouse (eight mice/group) were semiquantitatively measured using an ImageJ image processing and analysis system. The percentage of damaged tubules (interstitial inflammation and fibrosis, tubular dilation, and cast formation) was graded from 0 to 3 as follows: 0, normal; 1, tubular lesion <25%; 2, 25–50% lesion; and 3, lesion >50%. Immunohistochemical staining was performed on the 4 μm kidney sections using an SP kit (ZSGB-BIO; OriGene Technologies, Inc., Beijing, China). The sections were stained overnight with antibodies against KIM1 (1:100, dilution, Novus Biologicals, Littleton, CO, United States), collagen IV, fibronectin, collagen I (1:300, dilution, Proteintech, Chicago, IL, United States), α-smooth muscle actin (α-SMA), E-cadherin, transforming growth factor B1 (TGFB1), and peroxisome proliferator activated receptor alpha (PPAR-α; 1:100 dilution, Abcam, Cambridge, United Kingdom), respectively, at 4°C. The sections were further incubated with biotinylated secondary antibody for 1 h. Labeling was visualized using 3,3-diaminobenzidine to produce a brown color. Staining was analyzed under light microscopy using two independent, blinded observers. The collected images were assessed by the National Institutes of Health Image J software (Lo et al., 2012) (http://rsb.info.nih.gov/ij/) (Shi et al., 2013). Mitochondrial morphology was assessed using transmission electron microscopy (TEM). Briefly, small cubes of kidney cortex were fixed in 2.5% glutaraldehyde solution for 24 h at 4°C, and mitochondria were detected by Kingmed medical Test center (Taiyuan, China). A transmission electron microscope (Hitachi, Tokyo, Japan) was used to examine and photograph the sections.

### Western Blotting Analysis

Renal tissues or HK-2 cell samples were lysed using radioimmunoprecipitation assay (RIPA) buffer. Mitochondrial proteins were extracted using a Mitochondrial/Cytoplasmic Protein Extraction Kit (Beyotime, Shanghai, China), according to the manufacturer’s instructions. Equal amounts of protein were separated using sodium dodecyl sulfate polyacrylamide gel electrophoresis and transferred to polyvinylidene fluoride membranes (Millipore, Billerica, MA, United States). The membrane was incubated with primary antibodies specific to KIM1 (1:1000 dilution, Novus), α-SMA, E-cadherin, TGFB1 (1:1000 dilution, Abcam), carnitine palmitoyltransferase 1A (CPT1), acyl-CoA oxidase 1 (ACOX1), cytochrome C (CYCS), AMPK and phosphorylated (p)-AMPK (1:1000 dilution, Cell Signaling Technology, Danvers, MA, United States), collagen I, and β-actin (1:1000 dilution, Proteintech) overnight at 4°C. The membranes were then incubated with goat anti-rabbit or mouse IgG horseradish peroxidase conjugate (1:10,000 dilution, Santa Cruz Biotechnology, Santa Cruz, CA, United States) and scanned using an Odyssey Fc System (LI-COR, Lincoln, NE, United States).

### Quantitative Real-Time Reverse Transcription-PCR Analysis

The total RNA and cDNA from HK-2 cells were prepared using TRIzol reagent (Invitrogen, Carlsbad, CA, United States) and a High-Capacity cDNA Reverse Transcription Kit (Thermo Fisher Scientific) according to the manufacturer’s instructions. Quantitative real-time polymerase chain reaction (qPCR) analyses were performed using SYBR Premix ExTaq (Takara Bio Inc. Dalian, China) and an Agilent Mx3000P QPCR System (Agilent, Palo Alto, CA, United States). Expression levels were normalized to that of 18s rRNA. The following primer sequences were used: Mouse *Cpt1*: sense 5-GTG​ACT​GGT​GGG​AGG​AAT​AC-3′ and antisense 5′-GAG​CAT​CTC​CAT​GGC​GTA​G-3′; mouse *Acox1*: sense 5′-CCT​GAT​TCA​GCA​AGG​TAG​GG-3′ and antisense 5′-TCG​CAG​ACC​CTG​AAG​AAA​TC-3′; mouse *Ppara* sense 5′-TGA​GGA​AGC​CGT​TCT​GTG​AC-3′ and antisense 5′-GCA​AAT​CCC​TGC​TCT​CCT​GT-3′; human *CPT1*: sense 5′-GGA​GAG​GAG​ACA​GAC​ACC​ATC​CA-3′ and antisense 5′-CAA​AAT​AGG​CCT​GAC​GAC​ACC​TG-3′; human *ACOX1*: sense 5′-TGT​CCT​ATT​TGA​ACG​ACC​TGC​CCA-3′ and antisense 5′-AGG​TTC​CAA​GCT​ACC​TCC​TTG​CTT-3′; human *PPARA*: sense 5′-AGA​AGC​TGT​CAC​CAC​AGT​AGC-3′ and antisense 5′-CGC​GTG​GAC​TCC​GTA​ATG​AT-3′;

### Mitochondrial Morphology, Membrane Potential (ΔΨm), and Reactive Oxygen Species Detection

Mitochondria were labeled by incubating living HK-2 cells with the fluorescent probe MitoTracker Red (250 nM, Invitrogen) for 30 min. After staining nuclei with 4′,6-diamidino-2-phenylindole (DAPI), the stained cells were visualized under a confocal microscope (Olympus FV 1000 Viewer). Kidney tissues from the cortex was resected, trimmed, immediately chilled, homogenized in phosphate buffered saline (PBS), and filtered to produce a single cell suspension to measure the ΔΨm and mitochondrial ROS (mtROS). Measurement of ΔΨm was determined using a JC-1 Mitochondrial Membrane Potential Detection Kit (Biotium, Fremont, CA, United States) as described previously (Tang and Lai, 2012). Briefly, HK-2 cells or a single cell suspension of the kidney cortex cells were incubated with 1.5 µM JC-1 dye for 30 min at 37°C, washed three times, and resuspended in PBS buffer. The fluorescence was measured using red (excitation 525 nm/emission 590 nm) and green (excitation 488 nm/emission 525 nm) wavelengths using a flow cytometer (BD Immunocytometry Systems, Franklin Lakes, NJ, United States). The data are represented as the ratio of red to green (590/520). Increased green fluorescence levels and

decreased red fluorescence levels indicated a potential collapse of the mitochondrial membrane. MtROS were detected using the specific mitochondria-targeted superoxide fluorescent probe, MitoSOX (Invitrogen). Pretreated cells were incubated with a 5 μM of MitoSOX™ Red reagent working solution at 37°C for 10 min and then washed three times with warm PBS buffer. The measurement of mtROS was performed using flow cytometry, and the data were analyzed using FlowJo software (FlowJo LLC, Ashland, OR, United States).

### ATP Detection

ATP contents were assessed using a bioluminescence assay kit (Beyotime), according to the manufacturer’s instructions.

### Staining of Lipid Droplets

Freshly prepared kidney tissues were embedded in Optimal cutting temperature (OCT) compound and sectioned at 12 μm for Oil red O staining. The sections were rinsed with isopropanol and stained with Oil red O (Sigma-Aldrich) working solution for 30 min according to the manufacturer’s instructions. Nuclei were counterstained with hematoxylin for 5 min and examined using light microscopy. For lipid droplet staining of HK-2 cells, BODIPY 493/503 reagent (Thermo Fisher, Waltham, MA, United States) was used according to the manufacturer’s instructions.

### Quantitative Detection of Triglycerides

For lipid extraction and triglyceride analysis, 100 mg of kidney tissues, or four to five million cells, were collected, broken by ultrasonication for 1 min, centrifuged at 8000 × *g* for 10 min at 4°C, and the supernatant was retained. The triglyceride content was measured according to the manufacturer’s instructions (Nanjing Jiancheng Bioengineering Institute, Nanjing, China).

### Extracellular Flux Assay

HK-2 cells were seeded at a density of 50,000–80,000 cells/well in specialized XF96 cell culture microplates (Seahorse Bioscience, Billerica, MA, United States). The cells were exposed to different conditions as required. Then, the medium was replaced with Seahorse running medium (XF base medium supplemented with 10% D-glucose, 100 mM pyruvate, and 200 mM glutamine for the mitochondrial stress test, or with 200 mM glutamine alone for the glycolysis stress test). Then, incubation was performed in a non-CO_2_ incubator for 60 min at 37°C. The basal oxygen consumption rate and extracellular acidification rate were recorded for 24 min, followed by performance of the mitochondrial stress test (1 μM oligomycin, 2 μM carbonyl cyanide-p-trifluoromethox- yphenyl-hydrazon (FCCP), and 0.5 μM rotenone/antimycin A) and glycolysis stress test (10 mM glucose, 1 μM oligomycin, and 50 mM 2-Deoxy-d-glucose (2-DG)). Subsequently, the cells were lysed in RIPA buffer and subjected to the Bradford protein assay (Bio-Rad, Hercules, CA, United States). The oxygen consumption rate (OCR) and extracellular acidification rate (ECAR) values were normalized by the protein values in each well.

### Statistical Analysis

Data were expressed as the mean ± SD. Differences between groups were analyzed for statistical significance using one-way analysis of variance (ANOVA), followed by a post-hoc test using the Tukey–Kramer method. All experiments were performed at least three times. A threshold P-value < 0.05 was considered significant.

## Results

### Effects of BBR on Physical and Biochemical Characteristics of Diabetic Mice

Table 1 shows the general characteristics of the mice at the end of the experiment. The ratios of kidney weight to body weight (KW/BW), food intake, water consumption, and urinary fluid excretion were significantly higher in the diabetic db/db mice than in the control db/m mice. BBR treatment decreased the KW/BW and urinary fluid excretion, while food intake and water consumption have no obvious changed. The biochemical parameters were tested in each group of mice. BBR potently improved the urinary albumin excretion (UAE) and Scr of DKD. The FBG, which was elevated significantly in db/db diabetic mice, was apparently controlled using 12 weeks of BBR treatment. BBR treatment decreased the FFA, TG, TC, and LDL cholesterol levels, compared with that in the db/db group, and further increased the HDL cholesterol content in serum. These data demonstrated that BBR ameliorated metabolic disorder and renal dysfunction during DKD.

**TABLE 1:** Change of characteristics in each group.

Parameters	db/m (*n* = 8)	db/m + BBR (*n* = 8)	db/db (*n* = 8)	db/db + BBR (*n* = 8)
kidney/body weight (mg/g)	3.21 ± 0.48	5.41 ± 0.63	6.01 ± 1.01*	4.53 ± 0.64
food intake (g/day)	4.01 ± 0.18	3.91 ± 0.23	7.41 ± 1.01^*^	6.73 ± 0.64
water consumption (g/day)	9.12 ± 0.81	10.11 ± 0.76	22.55 ± 4.01^*^	21.03 ± 2.32
urinary fluid excretion (ml)	1.04 ± 0.05	0.96 ± 0.04	15.4 ± 0.68^*^	13.66 ± 0.18^#^
urinary albumin excretion (μg·24 h^−1^)	6.71 ± 1.26	7.11 ± 1.13	298.37 ± 11.94^*^	108.14 ± 10.52^#^
serum creatinine (μmol·L^−1^)	21.49 ± 2.45	23.45 ± 1.35	48.41 ± 6.01^*^	32.42 ± 2.54^#^
fasting blood glucose (mmol·L^−1^)	6.32 ± 0.42	6.05 ± 0.51	20.91 ± 3.82^*^	14.23 ± 2.43^#^
free fatty acid (mmol·L^−1^)	279.4 ± 14.47	289.4 ± 11.21	613.6 ± 13.45^*^	421.4 ± 14.45^#^
triglyceride (mmol·L^−1^)	0.94 ± 0.11	1.02 ± 0.09	2.61 ± 0.24^*^	1.84 ± 0.25^#^
total cholesterol (mmol·L^−1^)	2.39 ± 0.12	2.45 ± 0.35	6.41 ± 0.51^*^	3.42 ± 0.94^#^
low density lipoprotein (mmol·L^−1^)	0.61 ± 0.05	0.59 ± 0.03	1.21 ± 0.09^*^	0.78 ± 0.04^#^
high density lipoprotein (mmol·L^−1^)	0.43 ± 0.03	0.49 ± 0.05	1.11 ± 0.26^*^	1.34 ± 0.14^#^

Notes: KW/BW, kidney weight/body weight; UAE, urine albumin excretion; Scr, serum creatinine; FBG, fasting blood glucose; FFA, free fatty acid; TG, triglyceride; TC, total cholesterol; LDL, low density lipoprotein cholesterol; HDL, high density lipoprotein cholesterol. Date are presented as the mean ± SEM (*n* = 6). *p < 0.01 versus db/m; #*p* < 0.05 versus db/db.

### Effects of BBR on Histopathological Alterations and Tubulointerstitial Injury of Diabetic Mice

**FIGURE 1:**
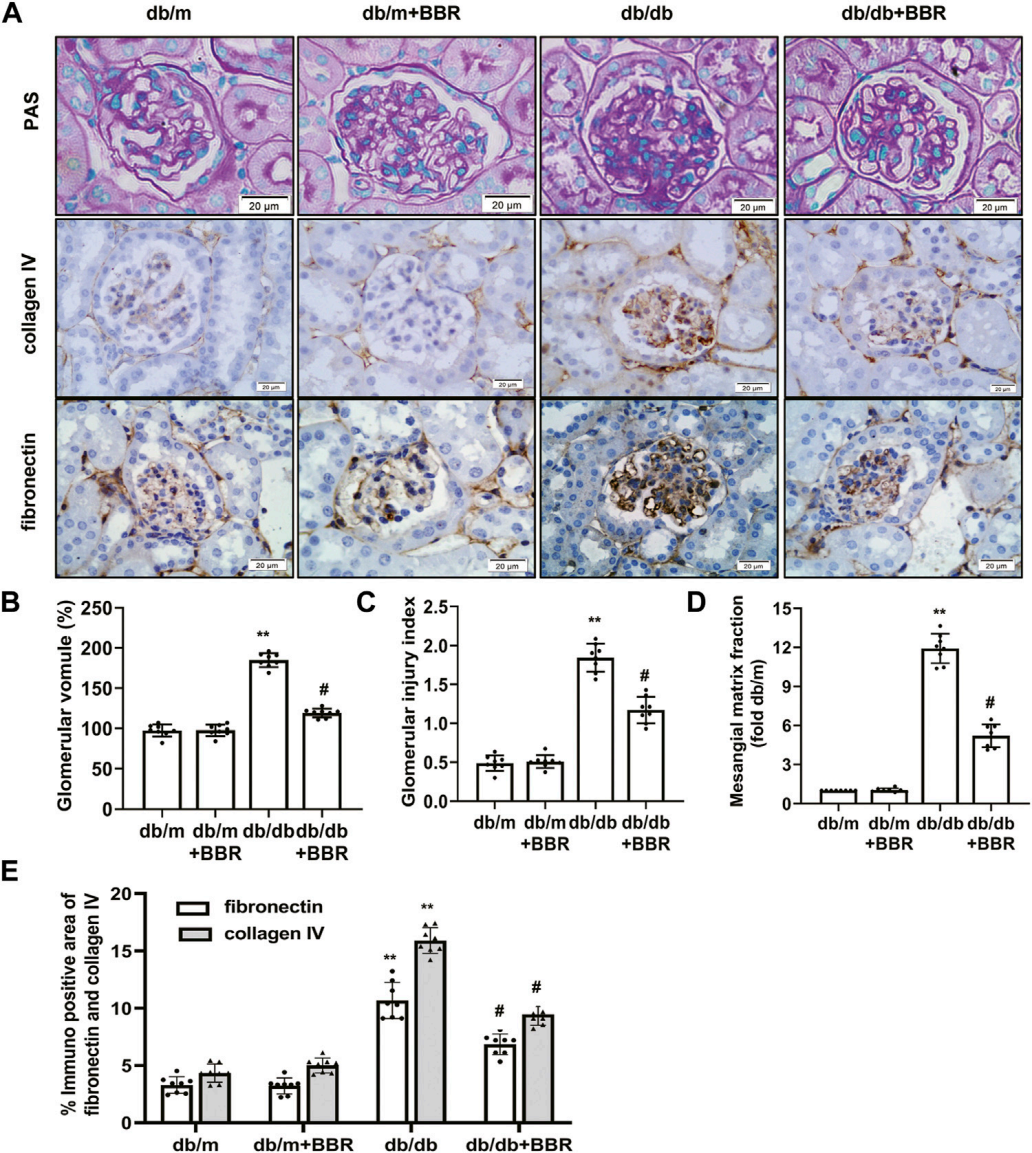
Effects of BBR on histopathological alterations of diabetic mice. **(A)** Representative photomicrographs of PAS staining and immunohistochemistry images of collagen IV, fibronectin (magnification 400×). **(B)** Semiquantitative analyses of the glomerular volume. **(C)** Semiquantitative analyses of the glomerular injury index. **(D)** Semiquantitative analyses of the mesangial matrix fraction. **(E)** Semiquantitative expression levels of collagen IV and fibronectin. db/m: normal mice + vehicle (0.9% saline of the same volume); db/m + BBR: db/m + BBR (300 mg/kg/d); db/db: diabetic mice + vehicle (0.9% saline of the same volume); db/db + BBR: db/db + BBR (300 mg/kg/d); (*n* = 8 mice/group). Data are expressed as means ± SD. **p < 0.01 versus the db/m group; #*p* < 0.05, compared with the db/db group by ANOVA.

We next evaluated whether BBR alleviated the histopathological alterations in kidneys of DKD. Compared with the db/m control mice, the accumulation of PAS-positive matrix increased significantly in the db/db diabetic mice. BBR treatment inhibited matrix expansion in kidney tissues (Figures 1A–D). In addition, fibronectin and collagen IV levels in the kidneys from db/db mice increased compared with those in the db/m group. Treatment with BBR decreased fibronectin and collagen IV expression significantly in diabetic db/db mice (Figures 1A,E). These results confirmed that renal injury in diabetic db/db mice is characterized by glomerular hypertrophy and mesangial matrix expansion, and that BBR could ameliorate the renal histopathological alterations in diabetic mice.

**FIGURE 2:**
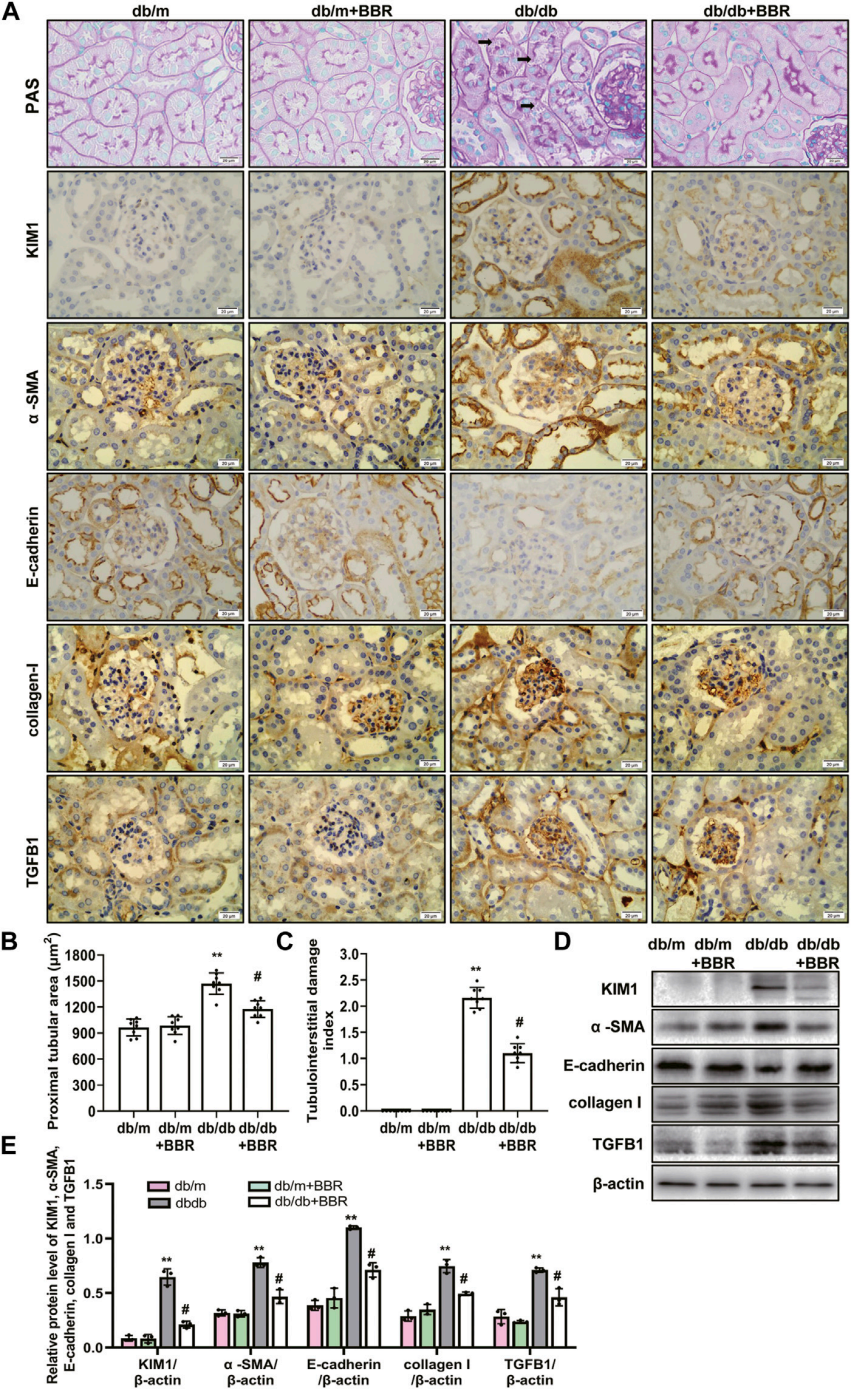
Effects of BBR on tubulointerstitial fibrosis of diabetic mice. **(A)** Representative photomicrographs of PAS staining and immunohistochemistry images of KIM1, α-SMA, E-cadherin, collagen I and TGFB1 (magnification: ×400). **(B)** Semiquantitative analyses of proximal tubular area according to the outer diameter. **(C)** Semiquantitative analyses of the tubulointerstitial damage index. **(D)** Representative western blotting images of KIM1, α -SMA, E-cadherin, collagen I and TGFB1. **(E)** Quantification of protein expression of KIM1, α -SMA, E-cadherin, collagen I and TGFB1. db/m: normal mice + vehicle (0.9% saline of the same volume); db/m + BBR: db/m + BBR (300 mg/kg/d); db/db: diabetic mice + vehicle (0.9% saline of the same volume); db/db + BBR: db/db + BBR (300 mg/kg/d); (*n* = 8 mice/group). Data are expressed as means ± SD. **p < 0.01 versus the db/m group; #*p* < 0.05, compared with the db/db group by ANOVA.

The recent finding supporting that proximal tubule and tubulointerstitial injury are important link in the earliest DN, and interacts with interstitial fibrosis create a vicious circle that promotes disease progression in diabetes (Russo et al., 2009; Tang and Lai, 2012; Zeni et al., 2017). Therefore, we next investigated the effects of BBR on tubulointerstitial lesions. PAS staining showed a increase in tubulointerstitial injury of db/db mice (Figure 2A). These pathological changes were attenuated by BBR treatment: The proximal tubular area (Figure 2B) and tubulointerstitial injury scores (Figure 2C) decreased significantly, whereas the normal control and BR control groups showed normal kidney tissue architecture. Since KIM1 is regarded as a hallmark biomarker of a proteinuric state and tubular damage, we then examined the expression of KIM1. Western blot analysis revealed a significant increase in KIM1 protein expression in the kidney of db/db mice compared with db/m mice and this was then decreased with addition of BBR. Immunohistochemistry staining showed that KIM1 was mainly expressed in tubular area. Immunohistochemistry staining for α -SMA, E-cadherin, collagen I, and TGFB1 were used to analyze the transdifferentiation of TECs (Figure 2A). The α -SMA, E-cadherin, collagen I, and TGFB1 were specifically localized in the interstitial compartment and the TECs of diabetic kidneys. However, α -SMA, E-cadherin, collagen I, and TGFB1 staining of the interstitial compartment and the TECs in the BBR-treated group was weaker than that in untreated diabetic db/db mice. Additionally, the protein levels measured using western blotting were consistent with the immunofluorescence staining (Figures 2D,E). Thus, BBR decreased tubulointerstitial injury in diabetic db/db mice.

### BBR Decreased Intracellular Lipid Accumulation and Increased the Expression of FAO Enzymes in the Proximal Tubules of Diabetic Kidneys

**FIGURE 3:**
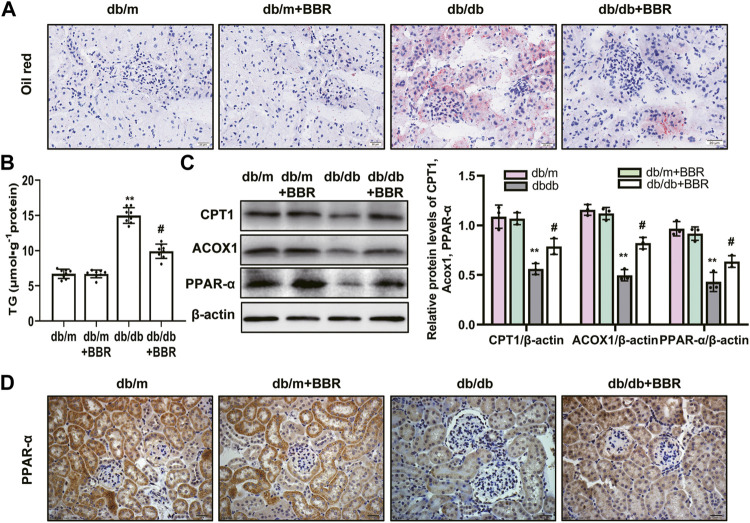
BBR decreased intracellular lipid accumulation and increased the expression of fatty acid oxidation enzymes in proximal tubules of diabetic kidney. **(A)** Lipid droplets accumulation was detected by Oil Red O staining (magnification: ×400). **(B)** The triglyceride levels of renal cortical tissues. **(C)** The expression level of CPT1, ACOX1 and PPAR-α in the renal cortex tissues was analyzed by western blotting. **(D)** Representative immunohistochemistry staining analysis of PPAR-α expression in the renal tissues of mice (magnification: ×400). db/m: normal mice + vehicle (0.9% saline of the same volume); db/m + BBR: db/m + BBR (300 mg/kg/d); db/db: diabetic mice + vehicle (0.9% saline of the same volume); db/db + BBR: db/db + BBR (300 mg/kg/d); (*n* = 8 mice/group). Data are expressed as means ± SD. **p < 0.01 versus the db/m group; #*p* < 0.05, compared with the db/db group by ANOVA.

Lipid droplet accumulation contributes to the development of tubulointerstitial injury in DKD; therefore, we examined BBR’s effects on the regulation of aberrant lipid accumulation in the kidneys using Oil Red O staining. The results revealed that lipid droplet accumulation was enhanced in the tubular cells of diabetic db/db mice, which was relieved by BBR treatment (Figure 3A). Diabetic db/db mice had markedly increased triglyceride levels in the renal cortex compared with those in non-diabetic db/m mice, and BBR decreased these TG levels (Figure 3B). The levels of enzymes and regulators of FAO are reduced in TECs, which contribute to intracellular lipid accumulation (Kang et al., 2015). To further evaluate the role of BBR in the regulation of FAO *in vivo*, we analyzed the expression levels of key FAO enzymes (CPT1, ACOX1, and PPAR-α) using western blotting and qRT-PCR. The expression changes in the levels of CPT1, ACOX1, and PPAR-α in diabetic db/db mice, which were downregulated significantly compared with those in db/m mice, were reversed by BBR treatment (Figure 3C). Furthermore, immunohistochemical staining revealed that PPAR-α was located in renal tubular cells. Compared with db/m mice, decreased positive PPAR-α signals were observed in diabetic db/db mice; and BBR treatment increased these signals in diabetic mice (Figure 3D). Thus, BBR might improve the DKD-impaired FAO of TECs.

### BBR Restored Mitochondrial Morphology, Mitochondrial Function, and Increased Mitochondrial Biogenesis in db/db Mice

**FIGURE 4:**
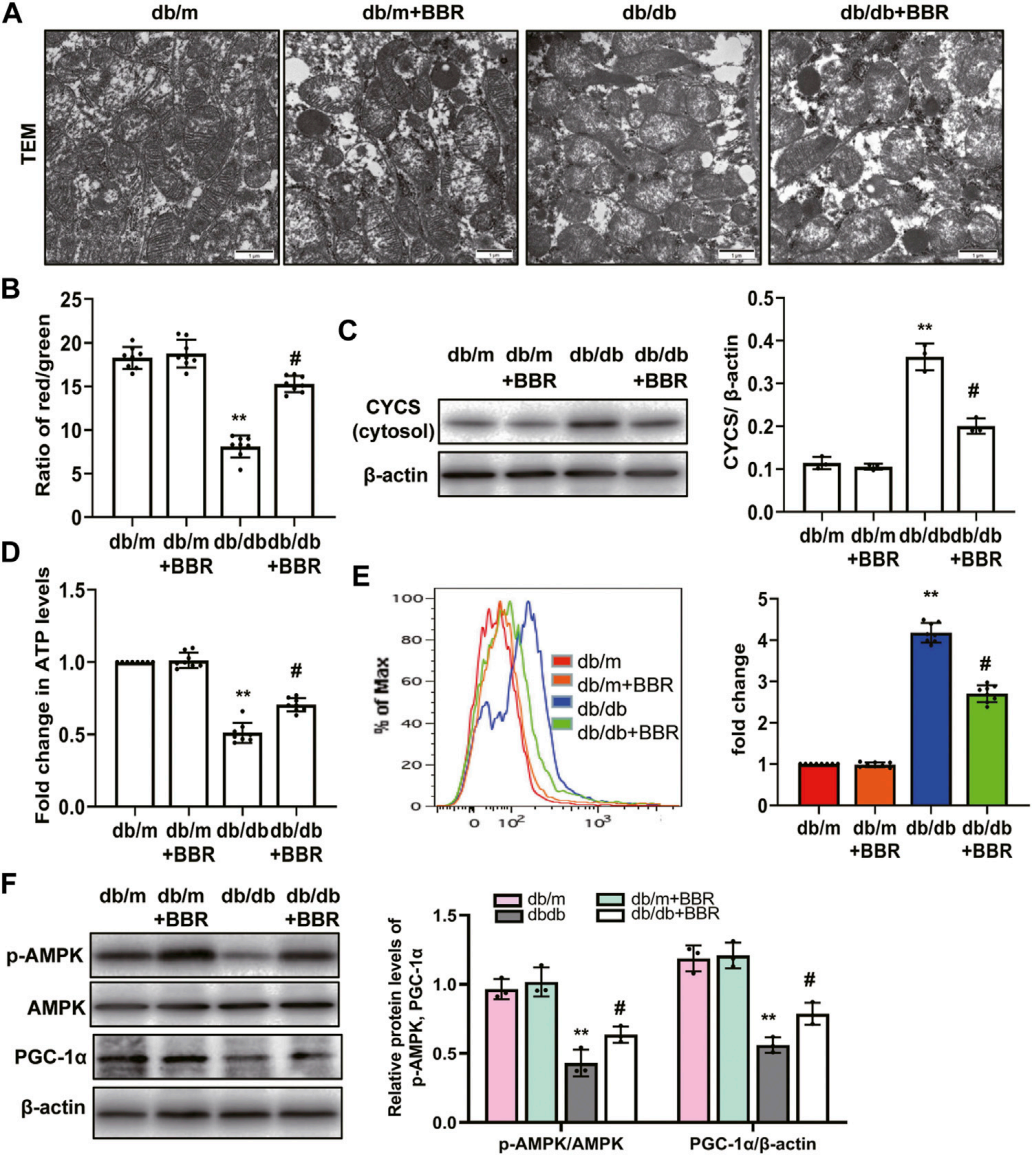
BBR restored mitochondrial morphology, mitochondrial function and increased mitochondrial biogenesis in db/db mice. **(A)** Mitochondrial morphology were examined by transmission electron microscopy (magnification: ×20,000). **(B)** mitochondrial membrane potentia (ΔΨ) was measured using a fluorescent probe JC-1. The ratio of red/green fluorescence represented ΔΨ m. **(C)** Subcellular extracts of CYCS was evaluated by western blotting analysis. **(D)** ATP levels were measured using an ATP bioluminescent assay kit. **(E)** Mitochondrial ROS was detected using MitoSOX by flow cytometry. **(F)** The expression levels of PGC-1α and p-AMPK were analyzed by western blotting and the relative intensity of PGC-1α and p-AMPK was normalized to the β-actin and total AMPK, respectively. db/m: normal mice + vehicle (0.9% saline of the same volume); db/m + BBR: db/m + BBR (300 mg/kg/d); db/db: diabetic mice + vehicle (0.9% saline of the same volume); db/db + BBR: db/db + BBR (300 mg/kg/d); (*n* = 8 mice/group). Data are expressed as means ± SD. **p < 0.01 versus the db/m group; #*p* < 0.05, compared with the db/db group by ANOVA.

Abnormal mitochondria are associated with lower FAO in TECs of DKD [10–813]. We next investigated the mitochondrial protective effects of BBR. TEM showed that TECs in db/m control mice contain numerous elongated mitochondria with a double membrane and cristae structure. By contrast, mitochondria in TECs of db/db mice were disorganized, with a low matrix density; BBR treatment partially reversed these changes (Figure 4A). To test whether BBR’s renal effects are linked to improved mitochondrial function and biogenesis of TECs *in vivo*, a single cell suspension of kidney tissues from the cortex (approximately 90% proximal tubules) of the mice in each group was prepared. Cells from db/db mice showed a low ΔΨm. Treatment with BBR reversed this alteration significantly (Figure 4B). Western blotting of subcellular extracts showed that CYCS levels in the cytosol fraction increased the in db/db mice and this increase was markedly attenuated by BBR treatment relative to that in the untreated db/db mice (Figure 4C). In addition, the changes in cellular ATP content mirrored those of the ΔΨ m. Higher ATP levels were found in the BBR-treated db/db mice relative to the untreated db/db mice (Figure 4D). A specific mitochondria targeted superoxide fluorescent probe, MitoSOX, was used to detected the mtROS. Flow cytometry analysis showed that there was a significant increase in red fluorescent signals in the kidneys of db/db mice compared with those in db/m mice. However, these levels were not as high in the BBR treated mice (Figure 4E). Mitochondrial biogenesis was assessed by measuring the expression of PGC-1α, a well-known regulator of the mitochondrial dynamics, and the activity of AMPK, which have profound effects on mitochondrial energy metabolism. BBR increased the levels of activated AMPK and PGC-1α in kidneys of diabetic db/db mice compared with those in the untreated db/db mice (Figure 4F).

### BBR Decreased Intracellular Lipid Accumulation and Increased the Expression of FAO Enzymes in HG-Induced HK-2 Cells

**FIGURE 5:**
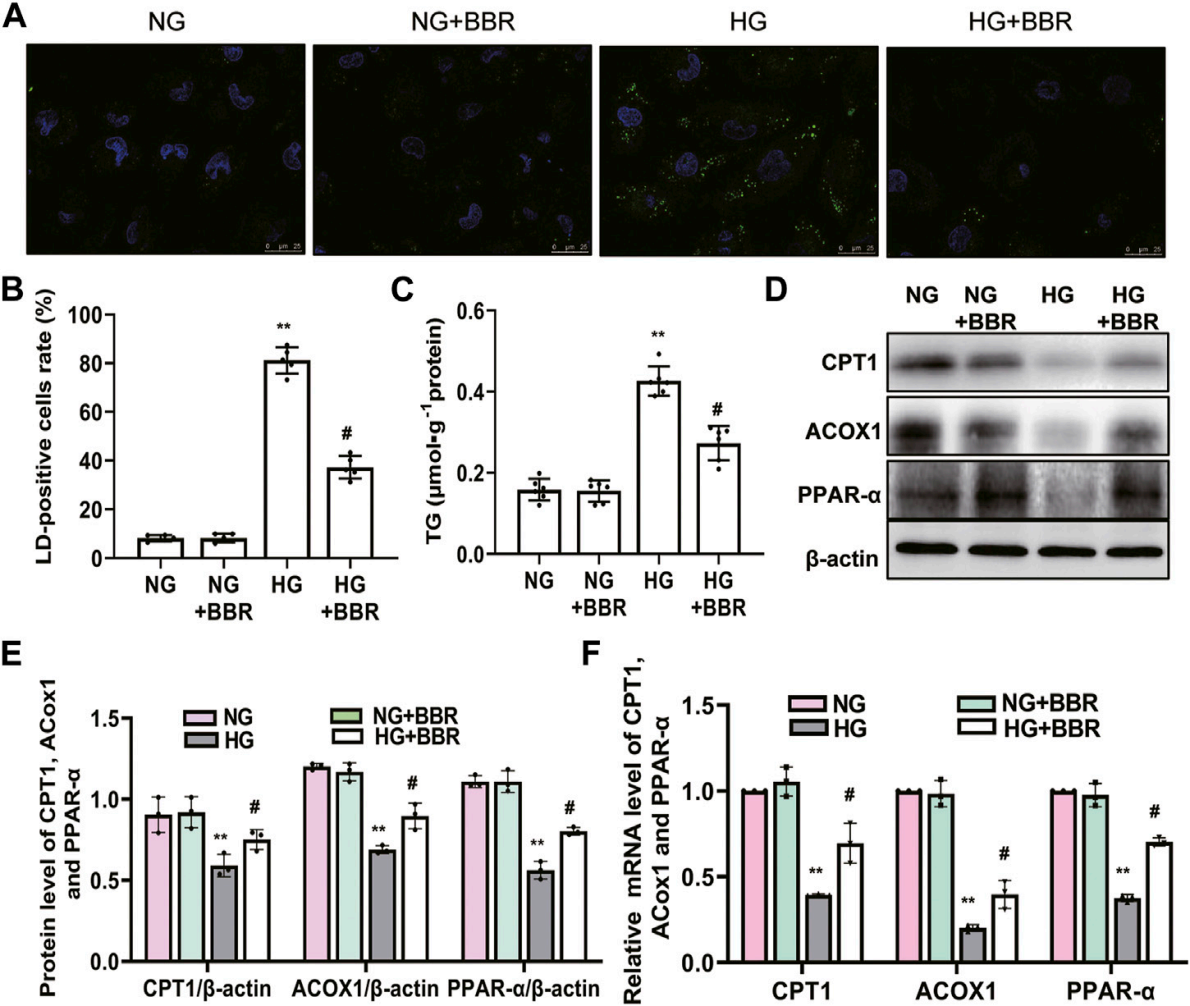
BBR decreased intracellular lipid accumulation and increases the expression of fatty acid oxidation enzymes in HG-induced HK-2 cells. **(A)** Lipid droplets (LD) accumulation was stained using BODIPY 493/503 and dvisualized under a confocal microscope. **(B)** Rate of LD-positive cells in cultured HK-2 cells. **(C)** The triglyceride levels of HK-2 cells. **(D,E)** The expression level of CPT1, ACOX1 and PPAR-α in HK-2 cells was analyzed by western blotting. **(F)** The mRNA level of CPT1, ACOX1 and PPAR-α in the renal cortex tissues was analyzed by RT-PCR. HK-2 cells were treated with 5.6 mM glucose (NG), NG+30 µM BBR (NG + BBR), 30 mM glucose (HG) or HG+30 µM BBR (HG + BBR) for 4 days; (*n* = 6). Data are expressed as means ± SD. **p < 0.01 versus the NG group; #*p* < 0.05, compared with the HG group by ANOVA.

To further detect and verify the protective effects and mechanism of BBR in renal TEC injury, we used HG-cultured HK-2 cells for *in vitro* experiments. HK-2 cells incubated with HG for 4 days showed increased green fluorescence staining of lipid droplets compared with those in the normal glucose (NG) group. BBR treatment decreased HG-induced intracellular lipid accumulation of HK-2 cells significantly (Figures 5A,B). Furthermore, the cellular triglyceride content in the HG group was higher than that in the NG group, which could be partially reversed by BBR treatment (Figure 5C). Next, we investigated the effect of BBR on FAO enzymes in HG-cultured HK-2 cells. Compared with the control group, HG stimulation 4 days decreased CPT1, ACOX1 and PPAR-α protein and mRNA levels significantly; whereas BBR suppressed the HG-induced downregulation of these enzymes in HK-2 cells (Figures 5D–F).

### BBR Ameliorated the Metabolic Reprogramming Induced by HG in HK-2 Cells

**FIGURE 6:**
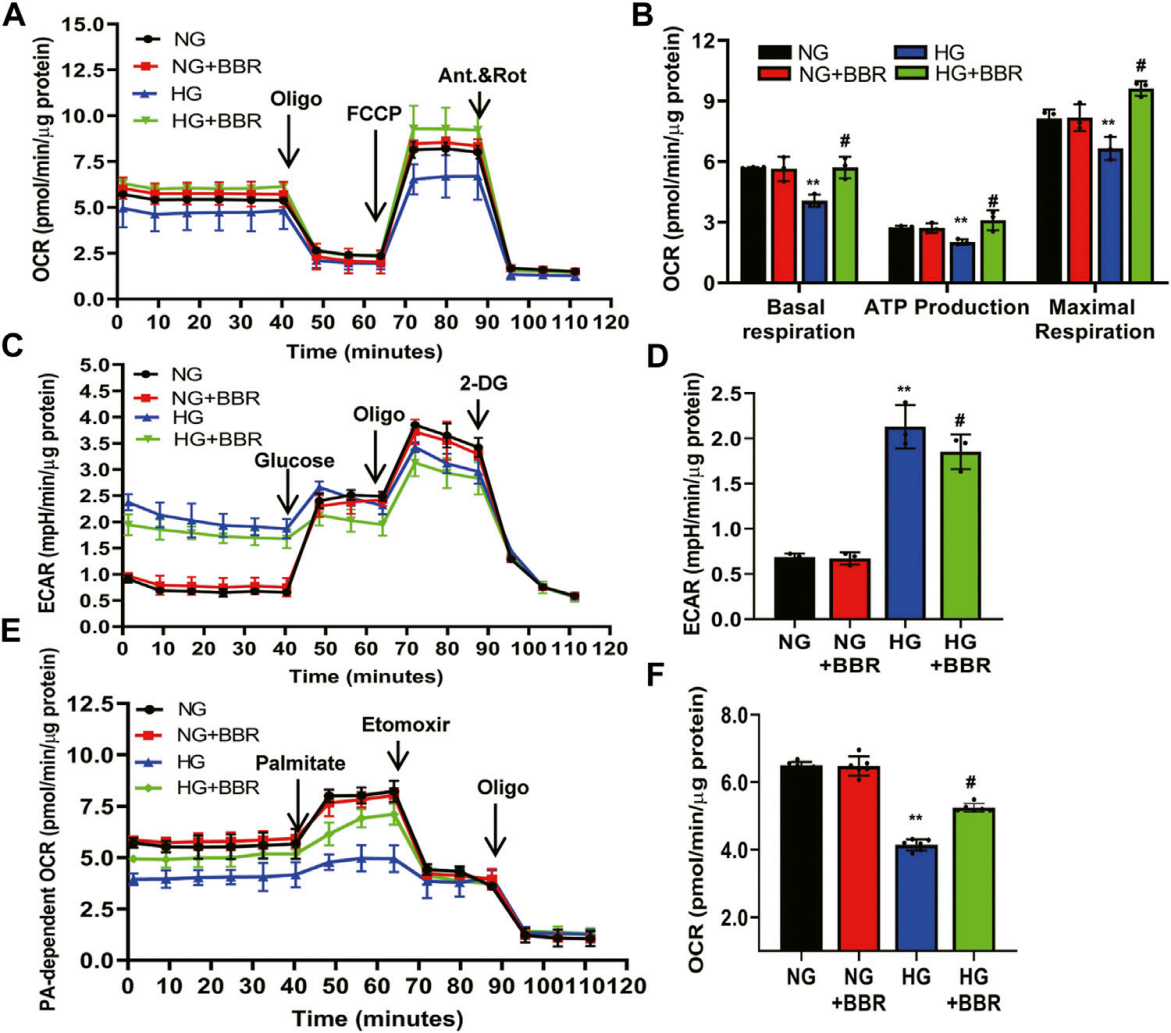
BBR ameliorates the metabolism reprogramming induced by HG in HK-2 cells. **(A–D)** The metabolic status of HK-2 cells was determined by evaluating the oxygen consumption rates (OCR) and the extracellular acidification rate of the media (ECAR) using the Agilent Seahorse XF technology. Left panels show representative OCR **(A)** and ECAR **(C)** curves from HK-2 cells treated with NG, M, HG, HG + BBR for 4 days. Right panels show quantified data from replicate studies **(B,D)**; *n* = 3 per group.**(E,F)** FA-driven oxygen consumption rate (PA-dependent OCR) curve **(E)** and OCR **(F)** quantified data from HK-2 cells. HK-2 cells were treated with 5.6 mM glucose (NG), NG+30 µM BBR (NG + BBR), 30 mM glucose (HG) or HG+30 µM BBR (HG + BBR) for 4 days; (*n* = 6). Data are expressed as means ± SD. **p < 0.01 versus the NG group; #*p* < 0.05, compared with the HG group by ANOVA.

Our *in vivo* data showed that BBR regulated FAO enzymes, suggesting that BBR reprograms TECs to use FAO under HG conditions. Thus, we next investigated the metabolic changes in HK-2 cells by treating them with NG and HG, with and without BBR, for 4 days, followed by a Seahorse extracellular flux analysis, which measures the OCR and ECAR. HG suppressed the OCR by ∼35% compared with that in the NG group (Figure 6A). The level of basal respiration, ATP production, and maximal respiration decreased significantly following HG treatment (Figure 6B). These results revealed an inhibition of mitochondrial oxidative phosphorylation. HG stimulation for 4 days also elevated the ECAR, which was consistent with a metabolic switch to glycolysis compared with the NG group (Figure 6C). Seahorse extracellular flux analysis in BBR-treated, HG-induced HK-2 cells showed a marked increase in the OCR (Figure 6B) and a decrease in the ECAR (Figure 6D), which was consistent with protection of mitochondrial health. These data indicated that BBR can cause cells rely on FAO to a greater extent under HG conditions. To directly demonstrate BBR’s effects on FAO, we measured the FA-driven oxygen consumption rate (palmitate-dependent OCR), using palmitic acid (PA) as a substrate. The OCR increased significantly in response to PA in NG-HK-2 cells, indicating that HK-2 cells efficiently metabolize PA (Figure 6E). BBR treatment of HK-2 cells was associated consistently with higher PA-dependent oxygen consumption under HG conditions compared with that in HG-induced HK-2 cells, which correlated with increased FAO (Figure 6F).

### BBR Restored Mitochondrial Morphology and Mitochondrial Function, and Increased Mitochondrial Biogenesis in HK-2 Cells

**FIGURE 7:**
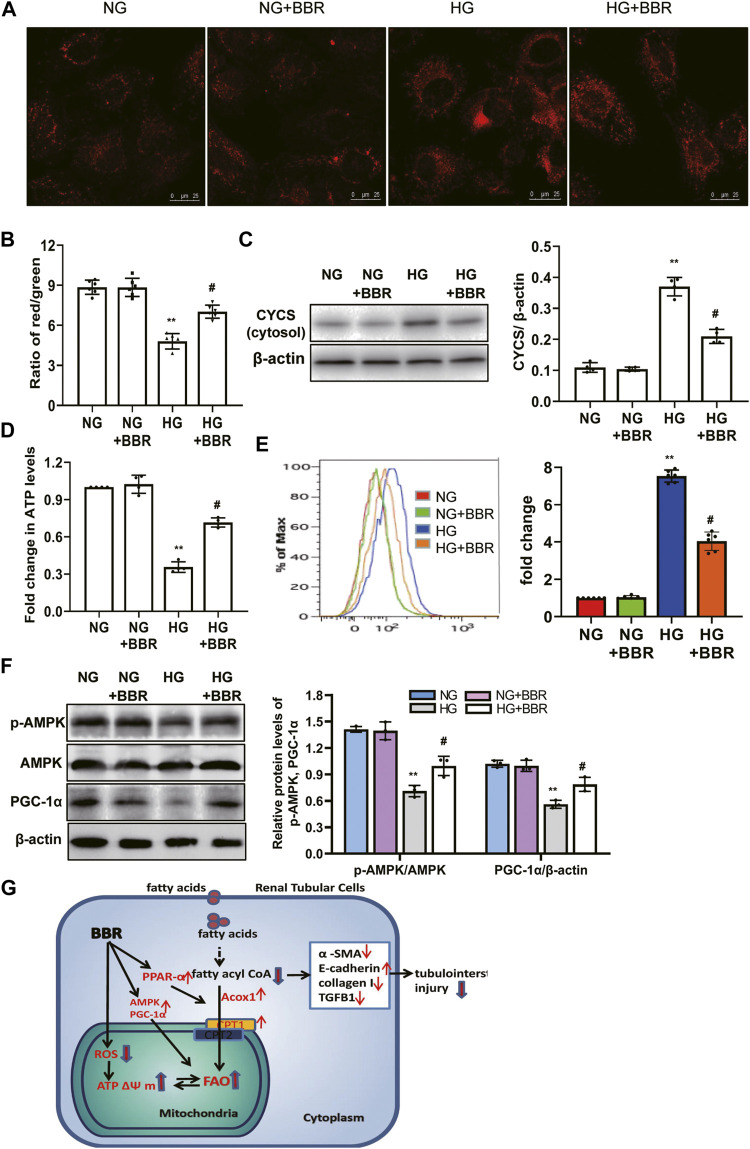
BBR restored mitochondrial morphology, mitochondrial function and increased mitochondrial biogenesis in HK-2 cells. **(A)** Mitochondrial morphology were stained by Mitotracker red and observed using confocal microscopy. **(B)** Mitochondrial membrane potential (ΔΨ) was measured using a fluorescent probe JC-1. The ratio of red/green fluorescence represented ΔΨ m. **(C)** Subcellular extracts of CYCS was evaluated by western blotting analysis. **(D)** ATP levels were measured using an ATP bioluminescent assay kit. **(E)** Mitochondrial ROS was detected using MitoSOX by flow cytometry. **(F)** The expression levels of PGC-1α and p-AMPK were analyzed by western blotting, and the relative intensity of PGC-1α and p-AMPK was normalized to the β-actin and total AMPK, respectively. **(G)**. A summary figure showing presumptive molecular targets of berberine in tubular epithelial cells of diabetes. HK-2 cells were treated with 5.6 mM glucose (NG), NG+30 µM BBR (NG + BBR), 30 mM glucose (HG) or HG+30 µM BBR (HG + BBR) for 4 days; (*n* = 6). Data are expressed as means ± SD. **p < 0.01 versus the NG group; #*p* < 0.05, compared with the HG group by ANOVA.

We investigated BBR’s effects on mitochondrial protection in HK-2 cells. To assess mitochondrial morphology *in vitro*, HK-2 cells were stained using Mitotracker red and observed using confocal microscopy. Compared with the NC group, the mitochondria in the HG group changed from a normal linear network to scattered fragments. This abnormal mitochondrial structure was alleviated by BBR treatment (Figure 7A). Furthermore, HK-2 cells incubated with HG for 4 days showed a low ΔΨ m. BBR treatment reversed this HG-induced alteration of ΔΨ m significantly in HK-2 cells (Figure 7B). Western blotting showed that HG treatment enhanced the release of Cytochrome C from the mitochondria into the cytosol. However, these changes were partially attenuated in BBR-treated HK-2 cells (Figure 7C). The changes in cellular ATP content mirrored those of the ΔΨm. BBR treatment also reversed the HG-induced alteration of ATP levels at 4 days (Figure 7D). Next, we evaluated BBR’s effect on mtROS production in HK-2 cells under HG conditions using MitoSOX: mtROS production in HK-2 cells increased significantly after HG stimulation for 4 days. Contrastingly, BBR treatment markedly suppressed mtROS production (Figure 7E). These data suggested that BBR restored mitochondrial function. Next, we evaluated the effect of BBR on HG-induced PGC-1α expression and AMPK activity. After HG treatment for 4 days, PGC-1α and p-AMPK levels decreased significantly (Figure 7F), whereas BBR treatment reversed this reduction.

## Discussion

The present study demonstrated that BBR treatment not only markedly relieved glomerular sclerosis, but also attenuated the tubulointerstitial injury in type 2 diabetic mice significantly. Moreover, mechanistic analysis showed that BBR could suppress TEC injury by improving HG-induced reduction of FAO, alleviating lipid deposition, and protecting mitochondria (Figure 7G).

BBR has known anti-diabetic effects in humans and experimental animals, and has been used for thousands of years. BBR regulates glucose metabolism and alleviates insulin resistance in diabetes. Its anti-inflammation and anti-oxidative stress activities have been suggested to be responsible for its anti-diabetic effects (Ma et al., 2018). A previous study reported that BBR significantly improved the renal function of STZ-induced diabetic mice (Zhang et al., 2016). In this study, we used type 2 diabetic db/db mice to observe the renoprotective effects of BBR. Our findings were consistent with previous findings that BBR regulates metabolic abnormalities, including hyperglycemia and dyslipidemia in db/db mice. BBR could decrease renal function-related parameters and improve the general state of mice. Renal morphology showed that BBR reduced glomerular matrix and collagen deposition significantly, which are typical of diabetic nephropathy, in 20-week-old mice after intervention for 12 weeks. However, although glomerulosclerosis is a defined feature of DN, TECs and tubulointerstitial injury determine the rate of decline in renal function (Vallon and Thomson, 2020). Studies have confirmed that damage to renal TECs is important in the development of diabetic nephropathy (Opazo-Ríos et al., 2020). However, little is known about the role of BBR in TEC injury in DKD. The present study showed that BBR also decreased tubulointerstitial injury scores and the proximal tubular area, improved tubular injury and epithelial-mesenchymal transition. These results suggested that BBR protects against diabetic nephropathy partly by protecting renal tubules.

Patients with type 2 DKD often present with dyslipidemia and metabolic disorders at both early and late stages (Hirano, 2018). In turn, lipid abnormalities accelerate DKD progression and the development of associated comorbidities (Kimmelstiel and Wilson, 1936). Renal TECs are more susceptible to damage by lipid metabolism disorders than other renal intrinsic cells because they obtain lipids not only from circulation, but also from the glomerular filtrate. In patients with diabetic nephropathy, the albumin concentration in urine is higher, and the filtration of albumin-bound fatty acids is increased, further aggravating renal tubular cell damage (Stadler et al., 2015; Hirano, 2018). In fact, in 1936, Kimmelstiel and Wilson first described the presence of lipid deposition in tubular cells of diabetic kidneys (Kimmelstiel and Wilson, 1936). Since then, a large body of evidence has shown that increased lipid deposition in renal tubule cells leads to lipid toxicity, causing inflammation of renal tubule cells and subsequent fibrosis. Hence, lipid-lowering therapy has become a beneficial therapeutic strategy for DKD. BBR played an effective role in regulating lipid metabolism in animal models. For example, BBR inhibited kidney damage in high-fat diet-fed rats (Wu et al., 2015). In addition, BBR protected hepatocytes in high-fat diet-fed mice by alleviating oxidative stress (Sun et al., 2017). Recently, BBR was observed to protect podocytes from injury and apoptosis induced by FFAs (Qin et al., 2019). Our results showed that in type 2 diabetes, BBR decreased TC, TG, and LDL-C levels, and increase HDL levels, indicating its effective role in reducing blood lipid. However, it is unclear whether BBR can inhibit lipid deposition in renal TECs. Our results showed that BBR inhibited lipid droplet formation in TECs from db/db mice and reduced cholesterol levels in cortical renal tissue. Intracellular lipid homeostasis is governed by a balance between fatty acid synthesis, absorption, and consumption; therefore, BBR’s effect of inhibiting renal tubular lipid deposition might be caused by the decrease of lipid uptake, followed by improvement of hyperlipidemia. However, BBR was observed to improve cellular FAO in renal tubule cells stimulated by PA and in podocytes induced by STZ in diabetic rats (Sun et al., 2018; Qin et al., 2020). The present study demonstrated that BBR upregulated FAO-related enzymes (CPT1, ACOX1, and PPAR-α) levels in db/db mice and HK-2 cells.

The kidney is a hypermetabolic organ, and under physiological conditions, about 90% of renal ATP is produced through oxidative phosphorylation in mitochondria. Most of the kidney-produced ATP is used to help the kidneys perform their physiological functions, such as actively reabsorbing sodium, glucose, ions, and other metabolites from filtered urine. These functions are completed by proximal renal TECs, thus playing an important role in renal metabolism. Metabolic changes are closely related to the normal function of the whole kidney. Physiologically, renal tubule cells mainly rely on mitochondrial FAO for energy. Evidence from 53 patients with CKD (including diabetes-induced CKD) and multiple animal models (Kang et al., 2015; Afshinnia et al., 2018; Console et al., 2020; Jang et al., 2020) showed that in advanced CKD, TEC metabolism changed from mitochondrial oxidative phosphorylation to glycolysis (the Warburg effect), and the oxidative damage to mitochondrial fatty acids aggravated the intercellular transdifferentiation and inflammation of TECs, and promoted fibrosis progression. Mitochondrial fatty acid metabolism restoration in TECs could improve interstitial lesions in CKD (Kang et al., 2015; Afshinnia et al., 2018). TEC metabolic reprogramming also occurs in acute kidney injury, promoting the progression from acute kidney injury to chronic interstitial injury (Jang et al., 2020). Reduced mitochondrial fatty acid oxidation increases lipid deposition and inflammation in renal tubules by disrupting the fatty acid balance (Kang et al., 2015; Afshinnia et al., 2018; Jang et al., 2020). Therefore, improving DKD tubular cell FAO is significant to treat renal tubular injury in diabetes. To further detect the effect of BBR on the metabolism of renal TECs *in vitro*, we used HG induced-HK-2 cells to detect metabolic change. High glucose stimulation of renal tubule cells for 4 days increased glycolysis and inhibited FAO, representing metabolic reprogramming. BBR intervention inhibited this change, and promoted renal tubule cell FAO. These results indicated that BBR inhibits lipid deposition in renal tubular cells of DKD partly by improving metabolic reprogramming.

Decreased FAO rates are closely related to mitochondrial dysfunction (Jang et al., 2020). Tubular cells have a high energy demand and a large number of mitochondria. Evidence shows that mitochondrial dysfunction plays an important role in renal TEC injury in diabetes mellitus (Ni et al., 2015; Viazzi et al., 2017). The present study showed that BBR administration could improve mitochondrial morphology of TECs in db/db mice and HK-2 cells. Mitochondrial damage may result in a low mitochondrial membrane potential, which causes the release of several proteins (e.g., cytochrome C) into the cytoplasm, consequently inducing cell apoptosis. Moreover, dysfunctional mitochondria are an important source of mtROS (Dan Dunn et al., 2015). Furthermore, ATP generation deficiency impairs cellular pumps, causing cell death (Shadel and Horvath, 2015). Our results revealed that BBR could alleviate all these mitochondrial damages and dysfunctions. AMPK is an important molecule in cellular energy homeostasis and metabolic pathways. As the crucial downstream signaling molecule of AMPK, transcriptional coactivator PGC-1α has been proved to be a master regulator of mitochondrial functions, including mitochondrial biogenesis, ΔΨ m, and mtROS production (Dan Dunn et al., 2015; Shadel and Horvath, 2015). We found BBR treatment enhanced the AMPK activation and promoted PGC-1α expression in TECs. This result indicated that BBR could improve mitochondrial function in TECs, and that mitochondria might be a target of BBR to alleviate metabolic disorders in patients with type 2 diabetes. However, the detailed mechanisms need to be confirmed by further experiments.

In conclusion, BBR alleviated TEC injury by improving HG-induced reduction of FAO, alleviating lipid deposition, and protecting mitochondria. We provide experimental evidence for the anti-DKD application of BBR *via* promoting FAO and reducing lipid accumulation in renal TECs.

## Data Availability

The raw data supporting the conclusion of this article will be made available by the authors, without undue reservation.
